# Calcium-phosphate complex increased during subchondral bone remodeling affects earlystage osteoarthritis

**DOI:** 10.1038/s41598-017-18946-y

**Published:** 2018-01-11

**Authors:** Youn-Kwan Jung, Min-Su Han, Hye-Ri Park, Eun-Ju Lee, Ji-Ae Jang, Gun-Woo Kim, Sun-Young Lee, DaeWon Moon, Seungwoo Han

**Affiliations:** 1 Laboratory for Arthritis and Bone Biology, Fatima Research Institute, Daegu Fatima Hospital, Daegu, Republic of Korea; 2 Division of Rheumatology, Department of Internal medicine, Daegu Fatima Hospital, Daegu, Republic of Korea; 3 Laboratory of Nanobio Imaging, Department of New Biology, Daegu Gyeongbuk Institute of Science and Technology (DGIST), Daegu, Republic of Korea

## Abstract

An activation of osteoclasts and subchondral bone remodeling is a major histologic feature of early-stage osteoarthritis (OA), which can be accompanied by an increase of calcium (Ca) and phosphate (Pi) level in the subchondral milieu. Considering articular cartilage gets most of nutrition from subchondral bone by diffusion, these micro-environmental changes in subchondral bone can affect the physiology of articular chondrocytes. Here, we have shown that Ca is increased and co-localized with Pi in articular cartilage of early-stage OA. The Ca-Pi complex increased the production of MMP-3 and MMP-13 in the hypertrophic chondrocytes, which was dependent on nuclear factor-kappa B (NF-kB), p38 and extracellular signal-regulated kinase (Erk) 1/2 mitogen-activated protein (MAP) kinase and Signal transducer and activator of transcription 3 (STAT3) signaling. The Ca-Pi complexes increased the expression of endocytosis markers, and the inhibition of the formation of the Ca-Pi complex ameliorated the Ca-Pi complex-mediated increases of MMPs expression in hypertrophic chondrocytes. Our data provide insight regarding the Ca-Pi complex as a potential catabolic mediator in the subchondral milieu and support the pathogenic role of subchondral bone in the early stages of cartilage degeneration.

## Introduction

Osteoarthritis (OA) is the most common type of degenerative joint arthritis and is characterized by cartilage loss and osteophyte formation^1^. OA was once thought to be a simple degenerative disease caused by excessive mechanical stress on articular cartilage. However, OA is currently known to have a complex etiology that affects the entire joint, and the production of matrix-degrading proteases plays a pivotal role^1,2^. Persistent mechanical stress induces a hypertrophic change in articular chondrocytes and the production of cartilage matrix-degrading proteases, such as matrix metalloprotease (MMP)-3, MMP-13, disintegrin and metalloproteinase with thrombospondin motifs 5 (Adamts5). Due to these changes, a central area in the cartilage undergoes cartilage loss, and the peripheral cartilage with a good blood supply from the synovium forms osteophytes through endochondral bone formation^2,3^.

Several studies investigating the pathogenesis of OA converge on the molecular cascades of producing cartilage matrix degrading proteases, such as MMP-13 and Adamts5^4^. In the producing these proteases, a lot of signaling mechanisms and transcription factors have been known to be involved as major upstream including nuclear factor-kappa B (NF-kB), mitogen-activated protein (MAP) kinases, hypoxia-induced factor 2 alpha (Hif-2α), and runt-related transcription factor 2 (Runx2)^4,5^. Among them, NF-kB signaling, which has known to play a role in biomechanical cartilage degeneration^6^, involves in the interleukin (IL)-1-mediated MMP-13 production and can strongly induce Hif-2α^7,8^. MAP kinases also participate in the induction of MMP-13 through extracellular signal-regulated kinase (Erk)-induced phosphorylation of ETS transcription factor, p38-mediated C/EBPβ and Runx2 activation, and c-Jun N-terminal kinase (JNK)-driven activator protein (AP)-1^9,10^. Because NF-kB and MAP kinases are major signaling molecules in the inflammatory response, inflammation in the synovium in response to cartilage breakdown products is believed to be a major upstream inducer of the NF-kB/MAP kinase-protease cascade^11^. In contrast to the inflammation hypothesis, several researchers have argued that OA is primarily a mechanical disease based on the role of misalignment or excessive loading during the development of OA^12^. However, the mechanism by which mechanical stress induces the activation of the NF-kB and MAP kinase cascades in OA remains unknown^13^.

In the context of mechanical loading, subchondral bone can be the primarily affected articular structure since subchondral bone is a major contributor to the dispersion of mechanical loading forces across the joint^14^. Indeed, activated osteoclasts are found in subchondral bone before the occurrence of the degradation of articular cartilage^15,16^. The subchondral bone pathology in early stage OA shows increased microfractures and a decreased thickness in the subchondral bone plate and trabecular bone, implying the presence of micro-damage and active remodeling of subchondral bone^17^. Therefore, the subchondral bone milieu in the early phase of OA may have a high concentration of calcium (Ca) and phosphate (Pi) due to resorption of the bone matrix by osteoclasts.

To date, most studies regarding the role of Ca and Pi in OA have focused on Ca-Pi crystals as a degradation product of bone involved in the late stages of OA^18^. Ca-Pi crystals, such as basic Ca-Pi, hydroxyapatite or Ca pyrophosphate dihydrate (CPPD), are increased in severe OA and can induce synovial inflammation^19,20^. Here, we hypothesized that the increased Ca-Pi levels in active subchondral bone remodeling can affect the physiology of articular cartilage in early stages of OA. To test our hypothesis, we applied Time-of-Flight Secondary Ion Mass Spectrometry (ToF-SIMS) to investigate the ion levels of Ca and Pi in articular cartilage. In addition, we analyzed the Ca-Pi complex-mediated global protease expression in hypertrophic chondrocytes, which revealed a significant increase in MMP-3 and MMP-13 expression that was dependent on NF-kB, p38 and Erk1/2 MAP kinase signaling. Our results provide insights into the mechanisms by which the changes in subchondral bone can affect articular cartilage degradation.

## Results

### Ca was increased and co-localized with Pi in articular cartilage in early-stage OA

**Figure 1:**
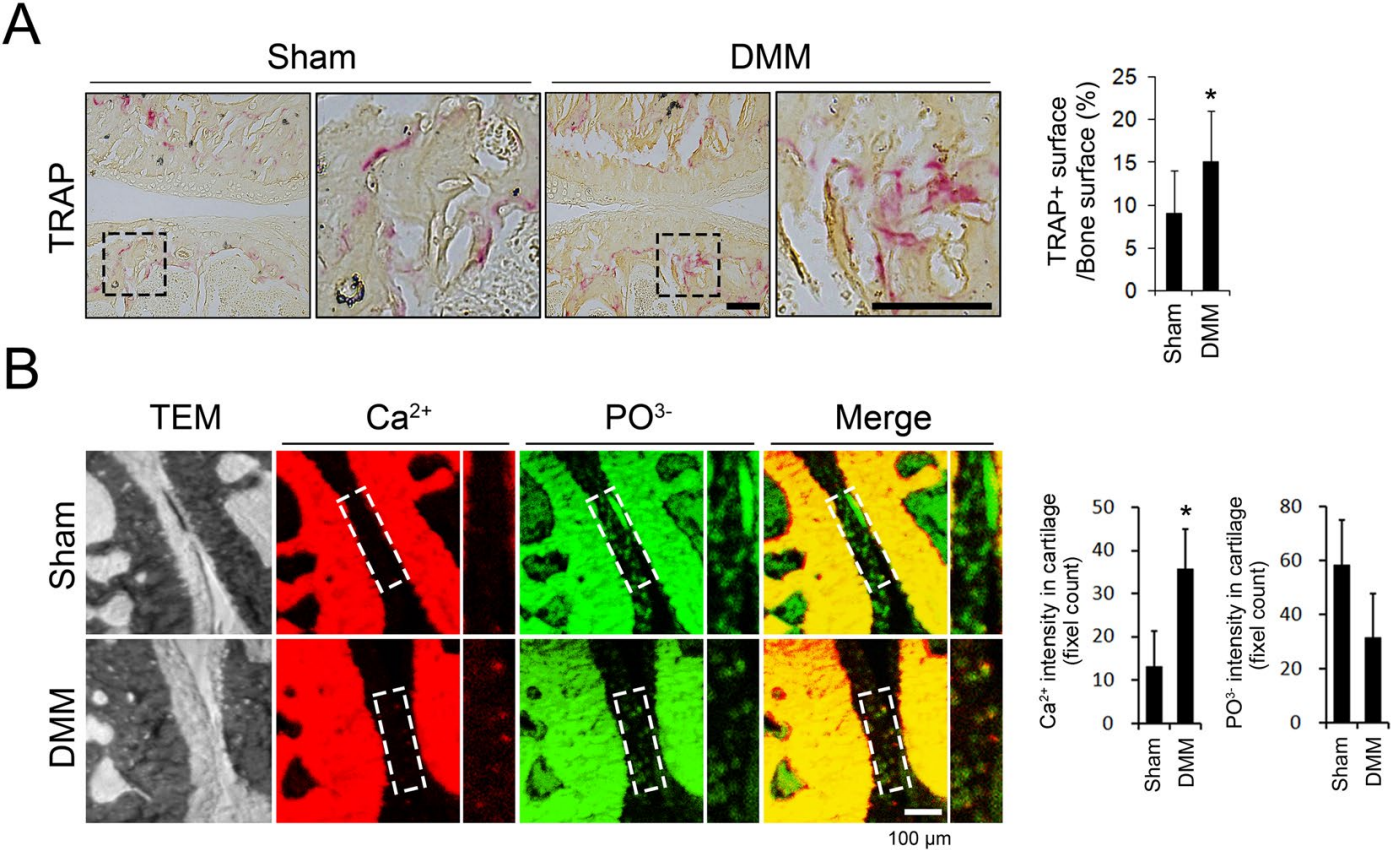
Ca^2+^ and PO_3_^−^ localization in articular cartilage following the DMM surgery. (**A**) Representative images of TRAP staining in subchondral bone 2 weeks after the DMM and control sham surgeries. Areas within the black rectangles are magnified in the right panels. TRAP-positive area per trabecular bone surface was quantified. Scale bar is 100 μm. (**B**) Representative images of ToF-SIMS analysis of the distribution of Ca^2+^ and PO_3_^−^ in articular cartilage. Original yellow colored images were converted to red for Ca^2+^ and green for PO_3_^−^ and then merged. Articular cartilage areas within the white rectangles are magnified in the right panels. The intensity of Ca^2+^ and PO_3_^−^ was quantified in multiple 47 × 47 μm-sized rectangles that were randomly selected in the area of the articular cartilage. TEM; Transmission electron microscopy. n = 7 in each group. *P < 0.05.

Evidence of increased subchondral bone remodeling in early OA prompted us to investigate whether Ca and Pi are increased in articular cartilage in OA^16^, which were quantified using ToF-SIMS analyses. The surgical DMM model was used to induce OA in 10-week-old male C57BL/6 J mice. As expected, compared to the sham-operated group, increased osteoclast activity, as assessed using TRAP staining, was observed as early as 2 weeks post-surgery in the subchondral bone area of the DMM group (Fig. 1A). The ToF-SIMS analysis of Ca^2+^ and PO_3_^−^ levels in articular cartilage revealed an increase in Ca^2+^ in the DMM surgery group, while PO_3_^−^ levels showed a decreasing trend in the DMM group compared to the those in the sham group. Interestingly, most Ca^2+^ in the articular cartilage in the DMM animals colocalized with PO_3_^−^, as is evident from the merge of the two channels producing yellowish punctuate signals. However, the articular cartilage in the control sham operation group remained green after merging the channels, indicating a predominance of PO_3_^−^ that was not colocalized with Ca^2+^ (Fig. 1B).

### Ca and Pi increase the expression of MMP-3 and MMP-13 in hypertrophic chondrocytes

Based on the Ca-Pi co-localization in the OA articular cartilage, we explored the effects of the Ca-Pi on catabolic enzyme production in hypertrophic chondrocytes since articular chondrocytes that are adjacent to calcified cartilage and subchondral bone, which are major sources of MMP-13, have hypertrophic features^21^. First, we assessed the Ca-Pi-mediated global changes in gene expression in hypertrophic chondrocytes obtained from 12-day micromass cultures of limb-bud MPCs. The microarray analysis revealed that the Ca (2.5 mM) and Pi (1.5 mM) treatment of the hypertrophic chondrocytes significantly increased the expression of MMP-3, MMP-13, and Adamts1 among the proteases involved in the proteolytic degradation of the extracellular matrix (ECM) (Supplementary Table 2).

**Figure 2:**
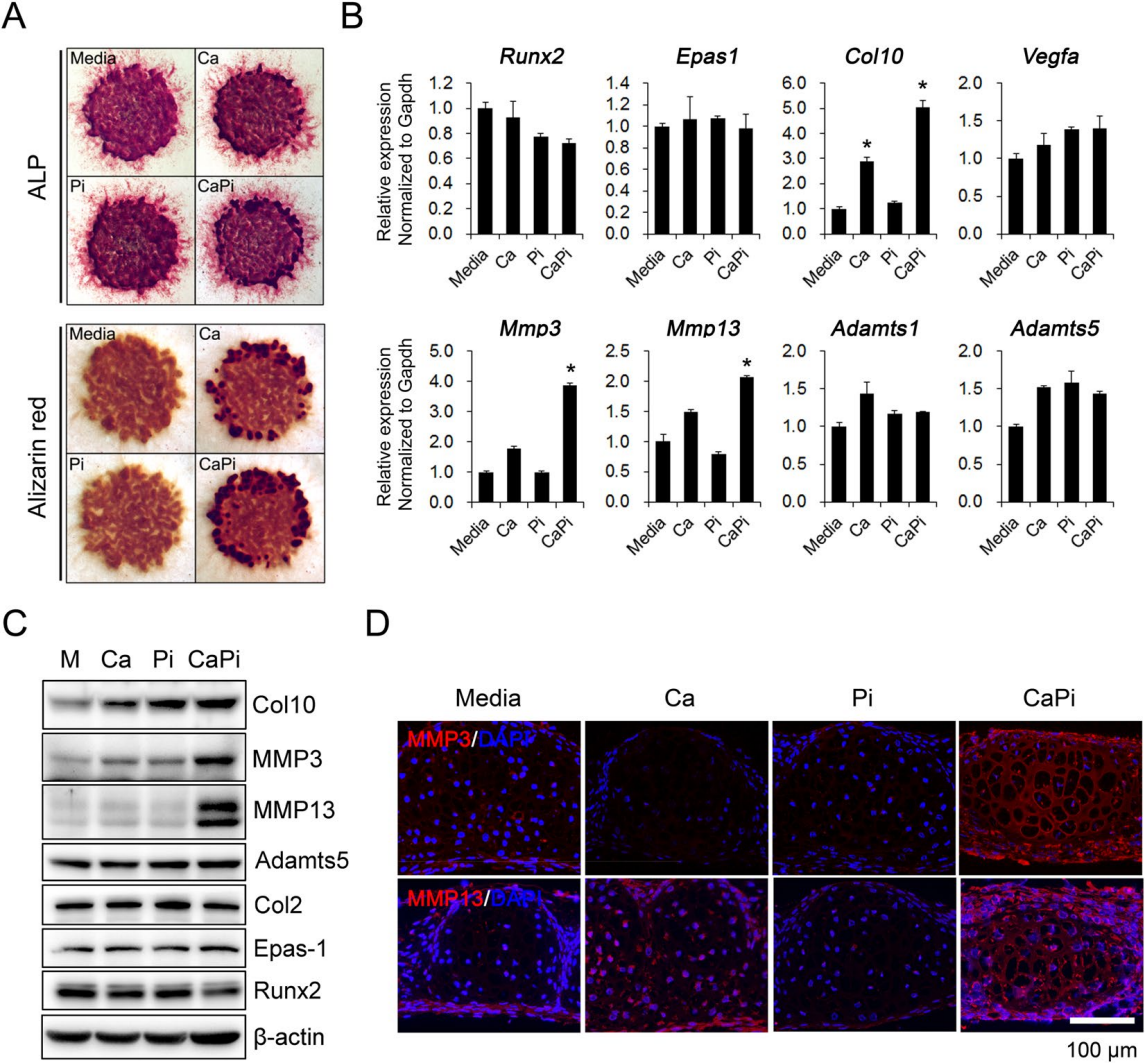
Ca- and Pi-mediated production of MMP-3 and MMP-13 in hypertrophic chondrocytes. (**A**) MPCs from E10.5 limb buds were high-density cultured (2.5 × 10^5^ cells/10 μL) for 12 days and treated with Ca (2.5 mM) or Pi (1.5 mM) for 1 day. Hypertrophic differentiation and matrix mineralization were assessed by ALP and Alizarin red staining, respectively. n = 5. (**B**) Gene expression related to the hypertrophic chondrocyte phenotype was assessed in Ca- or Pi-treated micromass cultures by real-time qPCR. (**C**) Total cell lysates from Ca- or Pi-treated micromass cultures were analyzed for chondrocyte differentiation markers by Western blotting. (**D**) Representative images of the expression of MMP-3 and MMP-13 assessed in Ca- or Pi-treated micromass cultures by immunostaining analysis. Scale bar = 100 μm. n = 5.

Expression of the identified targets was determined by real-time qPCR and a Western blot analysis of hypertrophic chondrocytes obtained from the micromass cultures (Fig. 2A). Ca and Pi increased the expression of type X collagen at the RNA level, while the expression of Runx2 was decreased, and Epas1 was not affected by the Ca and Pi treatment (Fig. 2B,C). As expected, MMP-3 and MMP-13 were significantly increased by the Ca and Pi treatment at the RNA and protein level, and this finding was confirmed by the immunostaining of the micromass cultures, which revealed a positive ECM staining of MMP-3 and MMP-13 following the Ca-Pi treatment (Fig. 2D). In contrast, the expression levels of Adamts1 and Adamts5 was unchanged following the Ca and Pi treatment (Fig. 2B,C).

### Ca-Pi-mediated MMP induction is dependent on Erk1/2, p38 MAP kinase, NF-kB and STAT3 signaling

**Figure 3:**
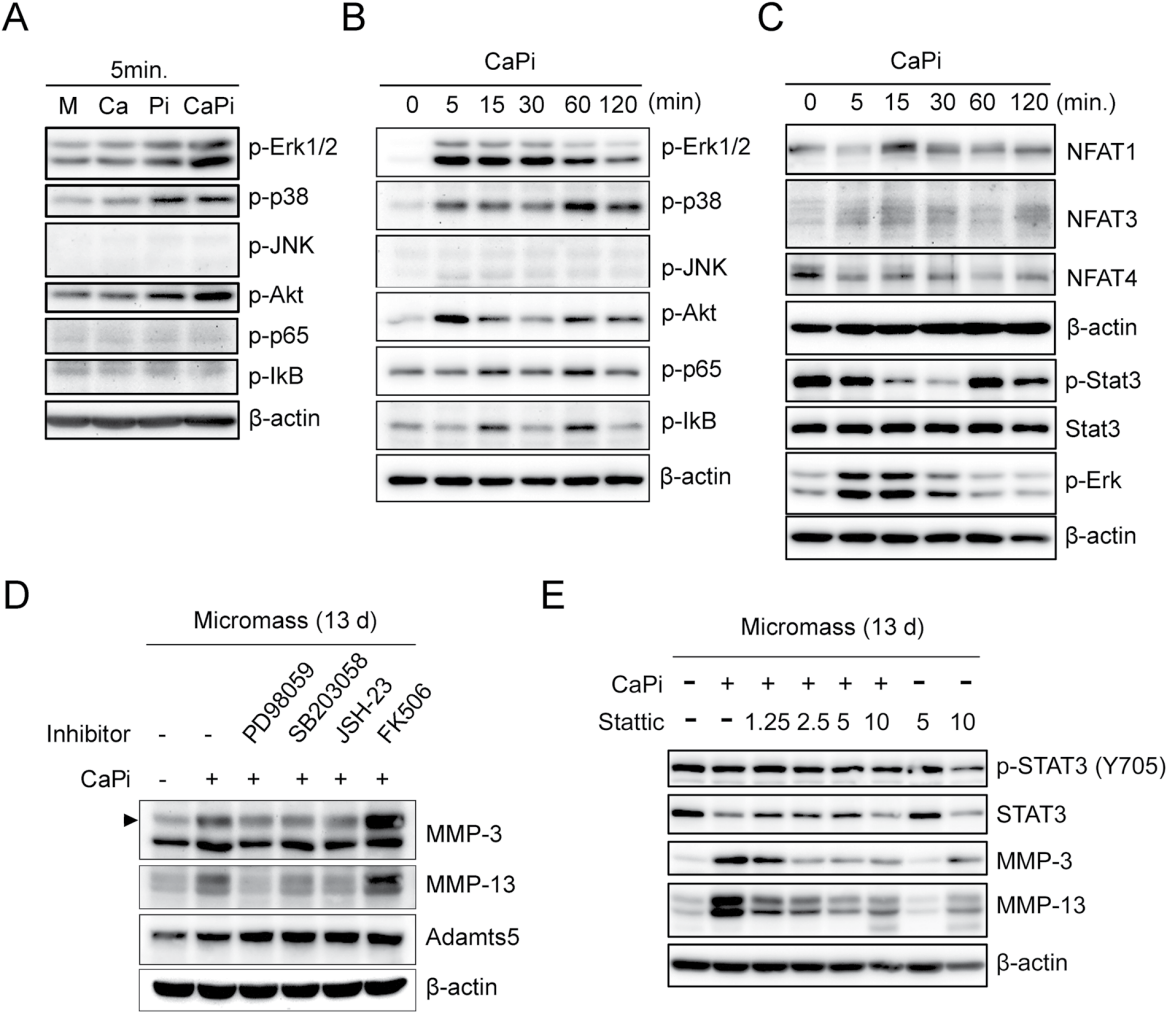
Ca- and Pi-induced activation of NF-kB, Erk1/2 and p38 MAP kinase, and STAT3 signaling is responsible for the MMP-3 and MMP-13 expression in hypertrophic chondrocytes. (**A**) Murine limb-bud MPCs were cultured at a high density (2.5 × 10^5^ cells/10 μL) for 12 days and then treated with Ca (2.5 mM) or Pi (1.5 mM) for 5 mins. Total cell lysates were subjected to Western blotting for Erk1/2, p38, JNK MAP kinase, Akt, and NF-kB signaling. (**B**,**C**) Hypertrophic chondrocytes obtained from micromass cultures were treated with Ca and Pi for the indicated time course, and the phosphorylation of the components in each signaling pathway was assessed by Western blotting. n = 3. (**D**) Representative Western blot analysis of whole cell lysates from micromass cultures treated with specific inhibitors of Erk1/2 (PD98059), p38 MAP kinase (SB203058), NF-kB (JSH-23) and calcineurin-NFAT signaling (FK506) during the co-treatment with Ca and Pi for 24 h. (**E**) Micromass-cultured cells were treated with various doses of Stattic, a small-molecule inhibitor of STAT3 activation and dimerization. n = 3.

To evaluate the transcriptional regulation of the Ca-Pi-mediated MMP production, we characterized the activation of the MAP kinases and NF-kB signaling, which has been previously identified as a key regulator of MMPs^22^. When we checked activation of these signalings at 5 min after Ca or Pi stimulation, the phosphorylation of Erk1/2, p38 MAP kinase and Akt signaling was increased by the Ca-Pi co-treatment and, to a lesser extent, the Pi treatment alone. At 5 min, the Ca or Pi treatment failed to activate JNK, MAP kinase, p65 and IkB, which are components of NF-kB signaling (Fig. 3A). However, a time course analysis of the changes in the signaling pathway following the Ca-Pi treatment revealed a delayed activation of p65 and IkB signaling at 15 min (Fig. 3B). We also measured changes in the NFAT family because these family members are major targets of Ca signaling. Of the members of this family, only NFAT1 was increased by the Ca-Pi stimulation at 15 min, which reflects the increased stability of this protein (Fig. 3C). We also checked the STAT3 signaling activation by Ca-Pi based on the recent data of basic Ca-Pi crystal-mediated IL-6-STAT3 signaling activation^19^. Interestingly, Ca-Pi treat initially reduced the phosphorylation of STAT3 and increased it in one hour, suggesting an indirect delayed stimulation of STAT3 (Fig. 3C).

We then investigated the role of these activated signaling pathways in MMP production by Ca-Pi. After blocking the Erk1/2, p38 MAP kinase and NF-kB signaling pathways with their specific inhibitors (PD98059, SB203058 and JSH-23, respectively), the Ca-Pi-mediated induction of MMP-3 and MMP-13 was significantly attenuated. However, the inhibition of the NFAT family by the calcineurin inhibitor, FK506 increased the production of MMP-3 and MMP-13, suggesting that the NFAT family plays a repressive role in MMPs transcription (Fig. 3D). The inhibition of STAT3 signaling with a various doses of Stattic, a small-molecule inhibitor of STAT3 activation and dimerization, significantly attenuated the Ca-Pi-mediated induction of MMP-3 and MMP-13 in hypertrophic chondrocytes (Fig. 3E).

### Ca-Pi complexes increase endocytosis in chondrocytes, and TLRs are not responsible for the MMPs production by Ca-Pi

**Figure 4:**
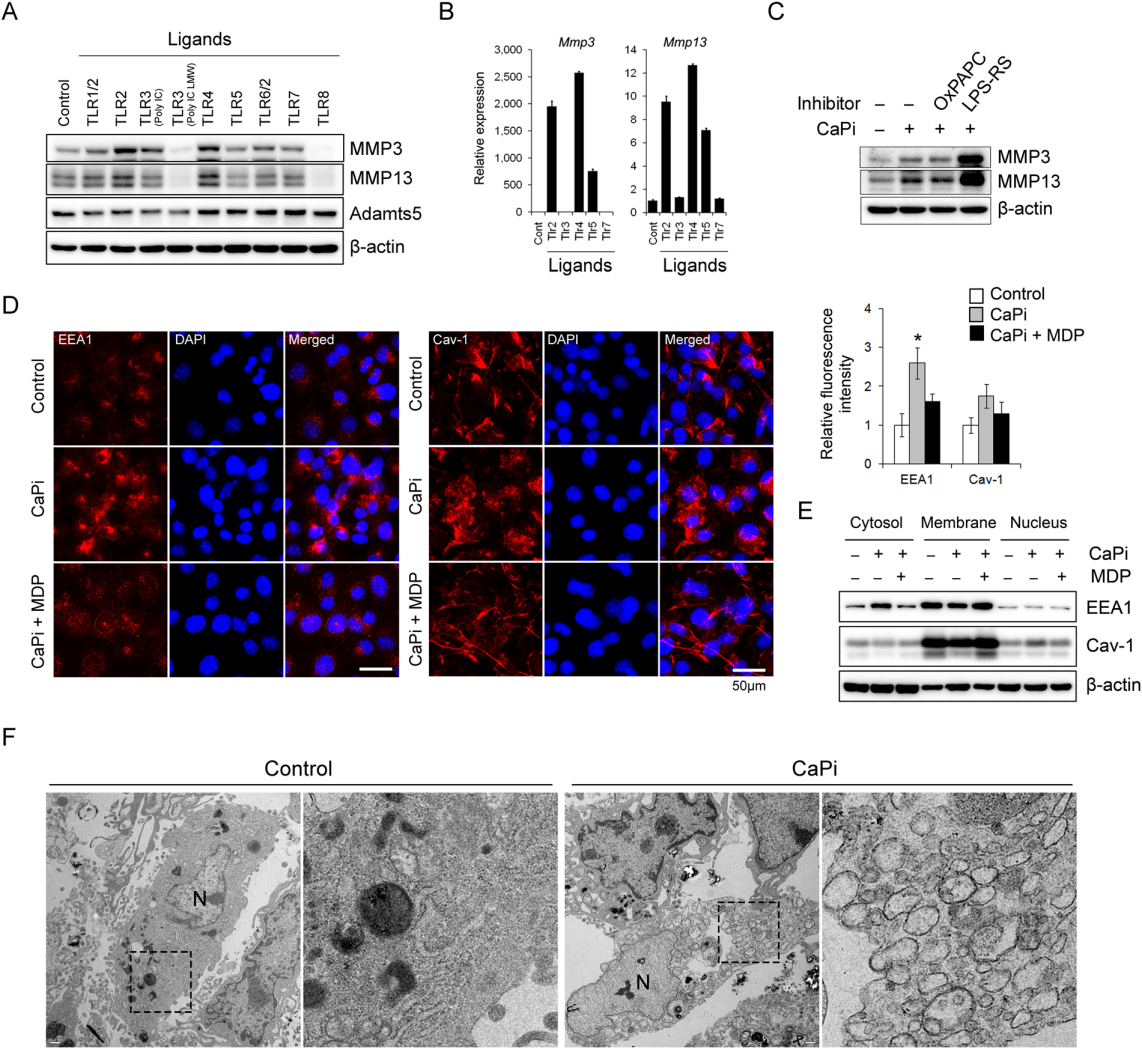
Ca and Pi increase endocytosis in chondrocytes, and their effects on MMP production may not be dependent on TLR signaling. (**A**,**B**) MMP-3, MMP-13 and Adamts5 protein and RNA production by TLR ligands were analyzed by Western blotting and real-time qPCR in hypertrophic chondrocytes obtained from 12-day high-density micromass cultures of limb-bud MPCs. (**C**) Representative Western blotting of MMP-3 and MMP-13 in Ca- and Pi-treated hypertrophic chondrocytes that were treated with 30 μM OxPAPC (TLR2 and TLR4 dual inhibitor) or 1 μM LPS-RS (TLR4-MD2 inhibitor). (**D**) Representative immunofluorescence staining of EEA 1, which is an early endosome marker, and Caveolin-1, a marker of matrix vesicles in primary chondrocytes in the presence of Ca-Pi or 1 mM MDP (a calcification inhibitor). Red fluorescence intensity was quantified using the ImageJ program. n = 8 per group. *P < 0.05. (**E**) Western blot analysis of subcellular fractionated lysates. Micromass-cultured hypertrophic chondrocytes were treated with Ca-Pi or MDP for 1 day. (**F**) Representative electron microphotography (EM) image of primary chondrocytes showing increased endosomes in the cytoplasm when treated with CaPi compared to controls. Images shown to the right is an enlargement of rectangle shown in the left panel. N represents the nucleus of chondrocyte.

To determine whether the Ca-Pi complex acts as a ligand for a specific receptor on chondrocytes, we investigated the activation of Toll-like receptors (TLRs), which are well-known receptors for Ca-containing crystals^23^. First, we determined the type of TLR signaling that is involved in the production of MMP-3 and MMP-13. When treated with various TLR ligands, the ligands for TLR2 and TLR4 both increased the expression of MMP-3 and MMP-13 at the protein and RNA levels (Fig. 4A,B). However, the pretreatment with OxPAPC, which is a dual inhibitor of TLR2 and TLR4, could not attenuate the increase in MMP-3 and MMP-13. Furthermore, LPS-RS, which is a TLR-4 antagonist that functions by binding MD2, rather increased the expression of MMP-3 and MMP-13. These results suggest that TLR signaling is not responsible for the Ca-Pi-mediated MMP induction (Fig. 4C). Ca-Pi crystals can be internalized into chondrocytes through endocytosis, which can increase the intracellular Ca levels and production of MMP and Adamts-family molecules^24,25^. Treatment of primary chondrocytes with Ca and Pi increased the expression of EEA 1, which is a marker of early endocytosis. In addition, the treatment increased the nuclear distribution of Caveolin-1, which is a major scaffolding protein in endosomes or matrix vesicles. However, treatment with methylene-diphosphonate (MDP), which is an inhibitor of the Ca-Pi complex formation that functions as an analog of pyrophosphate (PPi), completely restored the Ca-Pi-mediated changes in the endocytosis markers (Fig. 4D,E). To further clarify Ca-Pi-mediated enhanced endocytosis, we conducted electron microscophy (EM) analysis. Ca-Pi-treated primary chondrocytes revealed a prominent increase of endosome-like vacuoles in their cytoplasm compared to controls (Fig. 4F).

### The endocytosed Ca-Pi complex works as signaling effector

**Figure 5:**
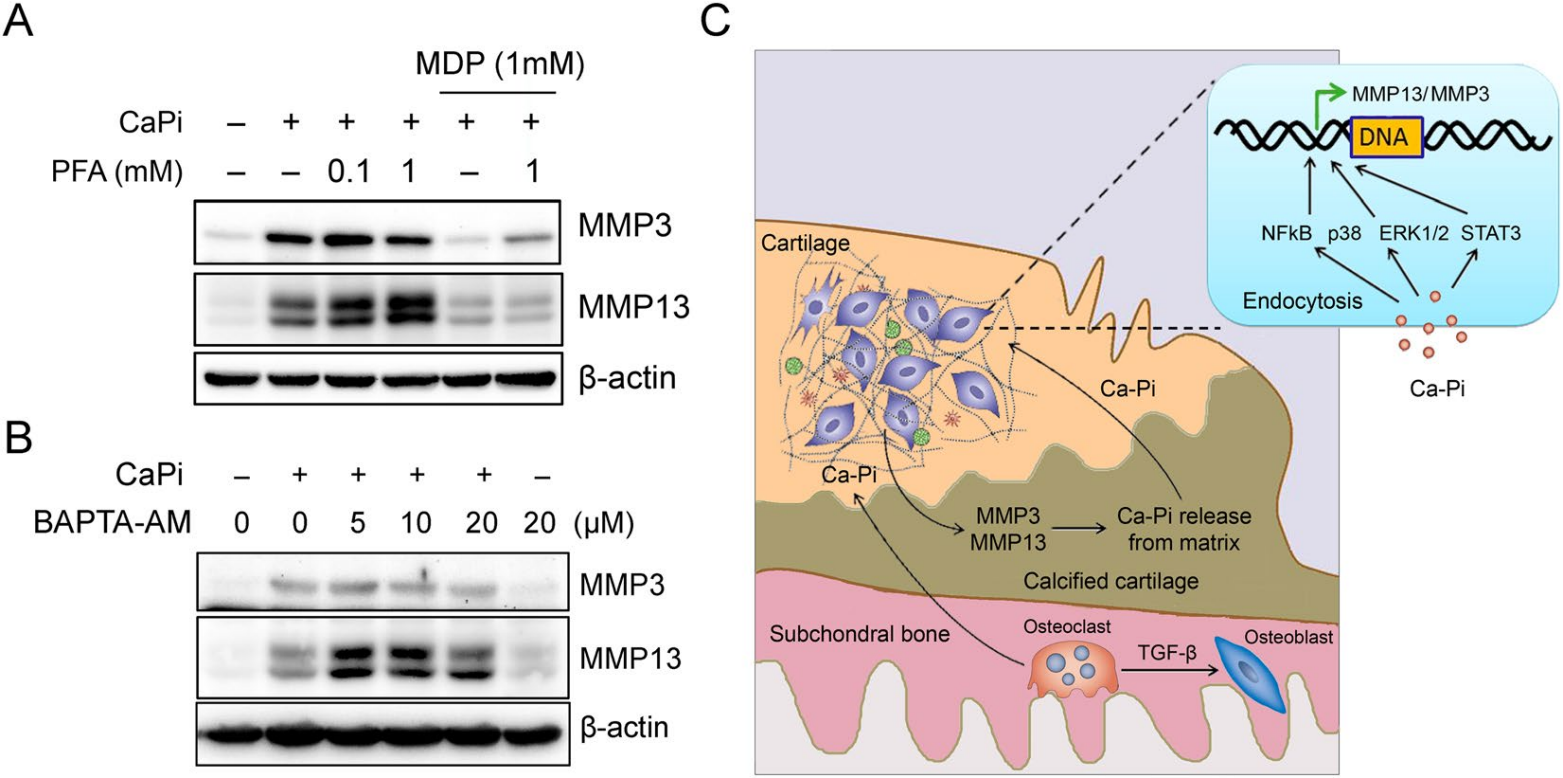
CaPi-mediated MMPs production depends on Ca-Pi complex formation (**A**) Representative Western blot analyses of MMP-3 and MMP-13 in high-density cultured hypertrophic chondrocytes. Cells were treated with Ca-Pi in the presence of PFA (a selective PiT1 and PiT2 inhibitor), or MDP (a calcification inhibitor) for 1 day. (**B**) Hypertrophic chondrocytes were treated with various doses of BAPTA-AM (an intracellular Ca chelator) with or without Ca-Pi for 1 day. (**C**) Schematic role of Ca and Pi in the pathogenesis of OA elicited from this study. Excessive mechanical stress can induce microfractures in subchondral bone. The microfractures can recruit macrophages to subchondral bone and promote bone remodeling. This remodeling can increase the Ca^+^ levels in articular cartilage, which primarily exists in a combined form with PO_3_^−^. The Ca-Pi complex induces MMP-3 and MMP-13 via the NF-kB, p38 and Erk1/2 MAP kinase, and STAT3 signaling pathways. The increased MMP-3 and MMP-13, in turn, release Ca and Pi from calcified cartilage through the degradation of the ECM. This positive catabolic loop associated with the Ca-Pi complex may accelerate the progression of OA.

To confirm whether the endocytosed Ca-Pi complex is responsible for the increase of MMPs, we treated the Ca-Pi formation inhibitor, MDP or phosphate channel inhibitor, phosphonoformic acid (PFA) in micromass-cultured hypertrophic chondrocytes with Ca and Pi. The inhibition of the Ca-Pi complex formation by MDP ameliorated the observed increase in the expression of MMP-3 and MMP-13 following the Ca-Pi treatment, whereas the inhibition of the Pi channels PiT1 and PiT2 with PFA failed to attenuate the observed increase in MMP expression following the Ca-Pi treatment (Fig. 5A). Finally, we investigated whether the intracellular signaling effects of Ca-Pi are mediated by free Ca ions. BAPTA-AM, which is an intracellular Ca chelator, failed to attenuate the Ca-Pi-mediated production of MMP-3 and MMP-13 in hypertrophic chondrocytes (Fig. 5B). Therefore, Ca-Pi can be endocytosed and act as a signaling effector in a complexed form.

## Discussion

Subchondral bone is considered a potential contributor to the pathogenesis of OA based on observations that subchondral microstructural damage and subchondral bone loss in the early stages of OA are strongly associated with cartilage damage^26–28^. Using a surgical model of OA, increased subchondral osteoclast activity was observed as early as 1 week after surgery before the articular cartilage damage occurred^16^. Therefore, molecules originating from subchondral bone in the remodeling process can affect the catabolic processing of articular cartilage in early OA. To the best of our knowledge, we are the first to identify an increase in the Ca ion content, which mainly exists in the form of a complex with Pi ions, in articular cartilage following surgically induced OA. Our data provide a model in which Ca, which is not normally present in articular cartilage, is increased, accompanied by increased subchondral bone remodeling during the early stages of OA. The increased Ca appears to mostly form a complex with Pi ions in the articular cartilage.

The Ca-Pi treatment of the hypertrophic chondrocytes significantly induces the production of MMP-3 and MMP-13 but not Adamts. This increase in MMPs was attenuated by a calcification inhibitor of a PPi analog but not by an intracellular Ca chelator or Pi channel inhibitor, suggesting that the Ca-Pi complex acts as a signaling molecule. Ca-Pi crystals have been known to act as mediators of inflammation in late stages of OA^18^. However, intra-articular injections of basic Ca-Pi crystals can induce OA-like changes^29^. In addition, Ca-Pi crystals have a positive feed forward loop with IL-6, which is an important mediator of joint damage as a key component of the senescence-associated secretory phenotype (SASP)^19^. We showed that the effects of Ca and Pi to hypertrophic chondrocytes including MMPs production and cellular changes all depended on Ca-Pi complex formation, but not by Ca or Pi ion alone. We used Ca or Pi-containing solution instead of Ca-Pi crystal, and did not characterize the exact molecular structure of Ca-Pi complex. Further study is needed to elucidate the functioning Ca-Pi complex structure as well as their signaling mechanisms.

MMP-3, which is most prominently induced in Ca-Pi-treated hypertrophic chondrocytes, is known to participate in the activation of additional latent MMPs, such as MMP-1 and MMP-13^30^. MMP-13 is the main collagenase involved in the degradation of type II collagen, which is sufficient to induce OA^31^. The cartilage of mice lacking MMP-13 was protected in a surgically induced OA model, while the cartilage-specific overexpression of MMP-13 aggravated spontaneous cartilage damage^32,33^. A spontaneous OA model of transgenic Del1 mice revealed that MMP-13 is expressed in deep calcified cartilage and subchondral bone where tissue remodeling is active but not in degenerative hyaline cartilage^21^. The expression of MMP-3 and MMP-13 in OA chondrocytes is known to be mainly regulated by Runx2 and Hif-2α^34,35^. However, in spite of the increase in MMP-3 and MMP-13, the expression of Runx2 and Hif-2α was surprisingly reduced following the Ca-Pi treatment in the hypertrophic chondrocytes, suggesting that the induction of MMPs by Ca-Pi is independent of Runx2 and Hif-2α.

Our results suggest that the Ca-Pi complex can activate NF-kB, p38 and Erk1/2 MAP kinase, and STAT3 signaling, and it was responsible for the production of MMP-3 and MMP-13 in hypertrophic chondrocytes. It is well known that the NF-kB and MAP kinase signaling are abnormally activated in OA chondrocytes, and these findings provided a foundation of inflammation theory in the development of OA^36,37^. There has been much debate regarding whether OA is a chronic inflammatory disease in which inflammatory cytokines from the synovium play a critical role, or a mechanically-induced “wear and tear” disease^38^. Here, our data provide a missing piece of the puzzle regarding how abnormal cartilage mechanics can be connected to the inflammation-related phenotype in OA as the Ca-Pi increases due to abnormal mechanics can activate NF-kB and MAP kinase signaling in hypertrophic chondrocytes.

BCP crystals are known to induce IL-6 production through involving Syk, PI3 kinases and STAT3 pathways in chondrocytes and IL-6, in turn, promotes BCP crystal formation, which leads to a positive amplification loop of BCP crystal formation^19^. In this study, the treatment of Ca and Pi gradually decreased the phosphorylation of STAT3 for the first 30 mins in hypertrophic chondrocytes. However, it showed a delayed activation in 60 mins after Ca-Pi stimulation, suggesting the presence of indirect trans-activation mechanisms in STAT3. In addition, the inhibition of STAT3 signaling with Stattic significantly attenuated the Ca-Pi-dependent production of MMPs in hypertrophic chondrocytes. This can be, same as the previous evidence^19^, dependent on the production of IL-6 by Ca-Pi complex. However, the early STAT3 activation just in one hour suggests the direct interaction with other signaling pathways such as NF-kB and MAP kinases. Our result provides another evidence of the interactions between Ca-Pi complex and STAT3 signaling pathway and further investigation is needed to elucidate the molecular mechanisms of Ca-Pi and STAT3 interaction.

From a clinical perspective, our data provide a theoretical basis for antiresorptive therapy to suppress bone remodeling and prevent the progression of OA. Although conflicting data have been reported, the NIH Osteoarthritis Initiative cohort study showed a trend for reduced knee joint space narrowing following bisphosphonate use after 4 years^39^. The benefits of antiresorptive agents in OA can depend on the timing of the treatment initiation, which may be the cause of conflicting clinical results. As subchondral bone remodeling is already active in the early stages of OA, the chondroprotective effects of antiresorptives can be maximized when used very early in the disease course. Antiresorptives have been shown to be effective in a surgical OA model using bisphosphonates immediately after the OA induction surgery^40^.

In conclusion, our data demonstrate for the first time an increase in Ca levels in articular cartilage during the early stages of OA and shed light on the role of the Ca-Pi complex as an intermediary that connects subchondral bone changes to articular cartilage (Fig. 5). These findings regarding the Ca-Pi- MMP feedback loop may introduce a new starting point for the development of disease-modifying OA drugs.

## Methods

### Surgical induction of OA and tissue preparation

Experimental OA was induced by destabilization the medial meniscus (DMM) as previously described^41^. Briefly, after anesthetizing 10-week-old C57BL/6 male mice, the left knee was dissected with a longitudinal incision approximately 3 mm in length over the distal patella to the proximal tibial plateau. The joint capsule was opened, and the infra-patella fat pad was dissected. The medial meniscotibial ligament, which is located at the end of the medial femoral condyle, was transected under a microscope to destabilize the medial meniscus. In the sham-operated mice, the medial meniscotibial ligament was visualized but not transected. The mice were maintained in their preoperative groups, allowed unrestricted cage exercise, and euthanized 2 weeks after the surgery. All animal protocols were approved by the Institutional Animal Care and Use Committee of the Daegu Fatima Hospital (approved protocol number F-15–03) and conformed to the Guide for the Care and Use of Laboratory Animals published by the US National Institutes of Health (NIH publication, 8^th^ Edition, 2011). The knee joints of the DMM or sham-operated mice were fixed in 4% paraformaldehyde (Merck, Darmstadt, Germany) at 4 °C for 24 h and dehydrated in increasing concentrations of ethanol for 12 days. Subsequently, the hydrated knee joints were embedded in methyl-methacrylate (MMA, Sigma) and sectioned at a thickness of 6 µm using a microtome with a tungsten carbide knife. The sections were mounted on glass slides for tartrate-resistant acid phosphatase (TRAP) staining or indium tin oxide (ITO) glass for ToF-SIMS analysis.

### TRAP staining and ToF-SIMS analyses

The engagement of osteoclasts in early OA was evaluated using TRAP staining after removing the MMA via 2-Methoxyethyl Acetate (AME, Sigma-Aldrich, St. Louis, MO) and rehydrating the sections. The rehydrated knee joint sections were incubated in a solution of naphthol-1-phosphate sodium salt and fast violet (Sigma-Aldrich) in an acetate buffer (pH 5.0) at 37 °C for 15 min. After washing with dH_2_O, the red-colored osteoclasts were analyzed under a microscope (Nikon, Japan).

The distribution of Ca^2+^ and PO_3_^−^ in the DMM and sham-operated knee joints was analyzed using a ToF-SIMS instrument (ION-TOF GmbH, Munster, Germany) equipped with a bismuth liquid metal ion gun (LMIG). A bismuth primary ion (Bi^3+^) beam at 30 keV in low-current bunched mode (pulse width ~1.13 ns, beam diameter ~2.0 μm) with a target current of 0.50 pA and a cycle time of 100 μsec was used to acquire the chemical images of the knee joints with a high-mass resolution. The primary ion dose density was 5 × 10^11^ ions∙cm^−2^. The analysis area was 500 × 500 μm^2^ (256 × 256 pixels) and was charge-compensated using an electron flooding gun. The positive and negative ion spectra were internally calibrated using Ca^2+^ and PO_3_^−^ ion peaks, respectively. To extract the ToF-SIMS spectra, 47 × 47 μm-sized regions of interest (ROIs) were manually selected in the cartilage region of the DMM or sham-operated knee joints. If multiple ROIs were selected in the same image, all ROIs were the same size, and their mass spectra were normalized to the total ion count. Each image was normalized to the intensity of the brightest pixel^42^.

### Micromass culture and inhibitor assay

The micromass culture was performed as previously described^43^. Briefly, limb buds from 10.5 dpc embryos were digested with Dispase II (Roche, Indianapolis, IN), and the suspended cells were grown in media composed of DMEM mixed with Ham’s F12 at a 2:3 ratio, 10% FBS, and 2 mM L-glutamine. The cells were concentrated at 2.5 × 10^7^ cells/ml, and a 10 μl drop was spotted on the culture plate. After achieving a stable attachment to the culture dish, the cells were differentiated into chondrocytes by a treatment with 10 mM β-glycerophosphate and 50 μg/ml ascorbic acid in the growth media and cultured for 12 days, and the media were changed every other day. To elucidate the role of the Ca-Pi complex in the hypertrophic chondrocytes, CaCl_2_ (2.5 mM) and a Pi buffer (1.5 mM) composed of Na_2_HPO_4_ and NaH_2_PO_4_-H_2_O were added to the 12-day-cultured micromasses for 24 h. For the inhibitor assays of each signaling pathway, the 12-day-cultured micromass cells remained untreated (control) or were stimulated with Ca and Pi for 24 h in the absence or presence of 50 μM PD98059 (MEK/Erk inhibitor), 10 μM SB203058 (p38 MAP kinase inhibitor), 20 μM JSH-23 (NF-kB nuclear translocation inhibitor), 10 μM FK506 (calcineurin inhibitor), various doses of Stattic (STAT3 inhibitor), 30 μM OxPAPC (TLR2 and TLR4 dual inhibitor), 1 μM LPS-RS (TLR4-MD2 inhibitor), 1 mM methylenediphosphonate (MDP; calcification inhibitor), various doses of phosphonoformic acid (PFA; selective PiT1 and PiT2 inhibitor) and BAPTA-AM (intracellular Ca^2+^ chelator).

### Microarray analysis

Total RNA was extracted from the micromass cultures that were treated with Ca-Pi or PBS as a control using an Easy-Blue™ Total RNA extraction kit (iNtRON biotechnology, Korea). After evaluating the RNA quality, 100 ng of total RNA were biotin-labeled using the Affymetrix GeneChip whole-transcript sense target-labeling assay and hybridized to Affymetrix mouse gene ST1.0 arrays (Affymetrix, High Wycombe, UK). After normalizing the scanned data using Expression Console software version 1.1 (Affymetrix), the differential gene expression was analyzed.

### Real-time qPCR analysis

Total RNA was extracted from the cultured primary chondrocytes or micromass cultures that were treated with Ca or Pi or untreated (control). In total, 2 μg of RNA were synthesized from cDNA and used to perform the real-time qPCR using the SYBR Green PCR master mix on an ABI ViiA7 real-time PCR machine (Applied Biosystems, Waltham, MA). All samples were run in triplicate and normalized to the expression of GAPDH. The calculation of the relative expression was performed using the 2-ddCT method. Each reaction was repeated three times independently. The primers used for the real-time qPCR are listed in Supplementary Table 1.

### Western blotting

Cell lysates from the micromass cultures or primary chondrocytes that were treated with Ca-Pi were separated by SDS-PAGE and transferred onto PVDF membranes (Amersham Biosciences, Buckinghamshire, UK). The membranes were blocked with 5% skim milk at room temperature (RT) for 1 h, followed by incubation with the primary antibodies listed below for 1 h with shaking. After washing the membrane with TBS-T, the membranes were incubated with an HRP-conjugated secondary antibody (Santa Cruz Biotechnology, CA, USA) for 1 h and washed again. The signal on the membranes was visualized using enhanced chemiluminescence solution (Amersham Biosciences). The primary antibodies against Col2, Col10, Mmp-3, Mmp-13, Adamts5 and Runx2 were purchased from Abcam (Cambridge, UK); the phospho-Erk1/2, phospho-P38, phospho-JNK, phospho-Akt, phospho-P65, phospho-IkB, NFAT1 and NFAT3 antibodies were purchased from Cell Signaling Technology (Danvers, MA); and the Epas1 and NFAT4 antibodies were purchased from Santa Cruz Biotechnology (Santa Cruz, CA).

### Immunofluorescence staining

The immunofluorescence staining was performed in cultured primary chondrocytes or sections of paraffin-embedded micromass samples. Primary chondrocytes cultured on 8-chamber slides (Thermo Fisher Scientific, Waltham, MA) were fixed with 4% paraformaldehyde (PFA) for 5 min and permeabilized on ice with 0.25% Triton X-100 in PBS for 3 min. After blocking with 2% BSA for 1 h, the cells were incubated with the anti-early endocytosis antigen (EEA1) (1:200) and anti-Caveolin-1 (1:200, Cell Signaling Technology, CA) antibodies at RT for 1 h. The cells were washed with PBS, incubated with an Alexa Fluor 594-conjugated anti-rabbit antibody (1:200, Thermo Fisher Scientific) for 1 h and counter-stained with DAPI.

For the immunostaining of the micromass cultures, paraffin-embedded micromass samples were cut into 3 µm-thick sections and then deparaffinized. After rehydration with decreasing concentrations of ethanol and antigen retrieval using proteinase K, the sections were blocked with 1% BSA for 1 h and incubated with the anti-Mmp3 and Mmp-13 antibodies (Abcam) in 1% BSA at 4 °C overnight. The sections were washed and incubated with Alexa Fluor 594-conjugated anti-rabbit antibody (1:200, Thermo Fisher Scientific) for 1 h. After counter-staining with DAPI, the sections were mounted with anti-fade mounting solution (Invitrogen, Carlsbad, CA) and imaged under a fluorescence microscope (Nikon, Japan).

### Electron microscopic analysis

Electron microscopy was performed to determine the number of endosome-like vacuoles in primary chondrocytes treated with or without Ca and Pi. Briefly, primary chondrocytes were cultured for 4 days and treated with or without Ca and Pi for 24 h, and then washed three times with 1X PBS, and collected by centrifugation at 1,000 × g for 5 min. The cell pellets were fixed in 4% paraformaldehyde overnight at 4 °C, post-fixed with 1% OsO4 in cacodylate buffer for 1 h at room temperature, and gradually dehydrated with ethanol. The dehydrated pellets were rinsed with propylene oxide for 30 min at room temperature and then embedded in Spurr resin prior to sectioning. Images of thin sections were observed under a transmission electron microscope (Tecnai G2 F20 TWIN TMP; FEI).

### Statistical analyses

The Mann-Whitney U tests and Kruskal–Wallis one-way analysis of variance (ANOVA) tests were used to determine the differences between the means. All analyses were conducted using SPSS version 14.0 software (SPSS, Chicago, IL). The results are presented as the means ± SDs, and statistical significance was accepted at P-values < 0.05.

## Electronic supplementary material

Supplementary Tables and Figures
