# Supplementary material for: Calcium-phosphate complex increased during subchondral bone remodeling affects earlystage osteoarthritis
Source: Sci Rep. 2018 Jan 11;8:487. doi: 10.1038/s41598-017-18946-y (PMC5765022; doi:10.1038/s41598-017-18946-y)

**Calcium-phosphate complex increased during subchondral bone remodeling affects early stage osteoarthritis**

**Youn-Kwan Jung^1^, Min-Su Han^1^, Hye-Ri Park^1^, Eun-Ju Lee^1^, Ji-Ae Jang^1^, Gun-Woo Kim^1,2^, Sun-Young Lee^3^, DaeWon Moon^3^ and Seungwoo Han^1,2^**

^1^Laboratory for Arthritis and Bone Biology, Fatima Research Institute, ^2^Division of Rheumatology, Department of Internal medicine, Daegu Fatima Hospital, Republic of Korea

^3^Laboratory of Nanobio Imaging, Department of New Biology, Daegu Gyeongbuk Institute of Science and Technology (DGIST), Republic of Korea

Supplementary Table 1. Primer sequences used for real-time PCR analysis

| Gene | Primer | Sequence 5' to 3' |
| --- | --- | --- |
| mRunx2 | Forward | GCCCAGGCGTATTTCAGA |
|  | Reverse | TGCCTGGCTCTTCTTACTGAG |
| mEpas1 | Forward | CTCCAGGAGCTCAAAAGGTG |
|  | Reverse | CAGGTAAGGCTCGAACGATG |
| mCol10 | Forward | GCATCTCCCAGCACCAGA |
|  | Reverse | CCATGAACCAGGGTCAAGAA |
| mVegfa | Forward | GCAGCTTGAGTTAAACGAACG |
|  | Reverse | GGTTCCCGAAACCCTGAG |
| mMMP3 | Forward | TTGTTCTTTGATGCAGTCAGC |
|  | Reverse | GATTTGCGCCAAAAGTGC |
| mMMP13 | Forward | GCCAGAACTTCCCAACCAT |
|  | Reverse | TCAGAGCCCAGAATTTTCTCC |
| mAdamts1 | Forward | GTGCAAGGGCAGTGTGTGAA |
|  | Reverse | CCACAAACGCCACACTTATCA |
| mAdamts5 | Forward | GCTGCTGGTAGCATCGTTACTG |
|  | Reverse | GTCCACGGTGCCTCTGTAGAA |

Supplementary table2. Microarray data for gene expression of cartilage matrix proteases by Ca and Pi in hypertrophic chondrocytes

|  |  | Fold Change | Normalized Data (log2) | |
| --- | --- | --- | --- | --- |
| Probe Set ID | GeneSymbol | Capi/Cont | Cont | Capi |
| 10583071 | Mmp3 | 9.165 | 7.56665 | 10.76278 |
| 10583044 | Mmp13 | 1.762 | 12.20575 | 13.02258 |
| 10367400 | Mmp19 | 1.202 | 7.854468 | 8.119555 |
| 10583056 | Mmp12 | 1.188 | 7.330598 | 7.579251 |
| 10389177 | Mmp28 | 1.155 | 7.499208 | 7.706823 |
| 10573924 | Mmp2 | 1.082 | 12.25727 | 12.37087 |
| 10503448 | Mmp16 | 1.06 | 9.262939 | 9.347481 |
| 10583112 | Mmp27 | 0.986 | 6.616653 | 6.596615 |
| 10583080 | Mmp1a | 0.963 | 4.800034 | 4.745354 |
| 10415052 | Mmp14 | 0.951 | 11.47204 | 11.40025 |
| 10583133 | Mmp7 | 0.935 | 6.055606 | 5.957982 |
| 10590781 | Mmp1b | 0.935 | 3.762239 | 3.664539 |
| 10370037 | Mmp11 | 0.927 | 9.154284 | 9.044321 |
| 10478633 | Mmp9 | 0.919 | 7.979093 | 7.856523 |
| 10568629 | Mmp21 | 0.902 | 5.664813 | 5.516686 |
| 10574350 | Mmp15 | 0.856 | 6.62172 | 6.397317 |
| 10477725 | Mmp24 | 0.828 | 6.403208 | 6.131268 |
| 10448278 | Mmp25 | 0.804 | 6.019533 | 5.704562 |
| 10583090 | Mmp10 | 0.794 | 6.392054 | 6.058901 |
| 10526038 | Mmp17 | 0.788 | 6.981262 | 6.637621 |
| 10583100 | Mmp8 | 0.751 | 5.380348 | 4.966517 |
| 10583122 | Mmp20 | 0.723 | 6.470589 | 6.003176 |
| 10519140 | Mmp23 | 0.649 | 10.45992 | 9.836979 |
| 10440522 | Adamts1 | 1.505 | 9.947671 | 10.53701 |
| 10440534 | Adamts5 | 1.161 | 10.04163 | 10.25692 |
| 10370798 | Adamtsl5 | 1.019 | 9.279981 | 9.307292 |
| 10531175 | Adamts3 | 0.953 | 7.341264 | 7.271884 |
| 10351551 | Adamts4 | 0.93 | 8.392939 | 8.287664 |
| 10369431 | Adamts14 | 0.925 | 7.817438 | 7.70539 |
| 10591988 | Adamts15 | 0.904 | 6.578378 | 6.432868 |
| 10375751 | Adamts2 | 0.87 | 9.560955 | 9.360012 |
| 10546432 | Adamts9 | 0.829 | 8.484278 | 8.212969 |
| 10531185 | Adamts3 | 0.819 | 8.648783 | 8.360159 |
| 10531187 | Adamts3 | 0.812 | 8.824068 | 8.524504 |
| 10410477 | Adamts16 | 0.805 | 6.436383 | 6.1235 |
| 10531177 | Adamts3 | 0.804 | 9.321782 | 9.006276 |
| 10587748 | Adamts7 | 0.798 | 7.766328 | 7.440982 |
| 10531181 | Adamts3 | 0.792 | 8.166506 | 7.829443 |
| 10531173 | Adamts3 | 0.783 | 8.394163 | 8.041067 |
| 10423109 | Adamts12 | 0.778 | 10.24085 | 9.87874 |
| 10406982 | Adamts6 | 0.767 | 9.061533 | 8.679039 |
| 10554599 | Adamtsl3 | 0.764 | 9.85334 | 9.464825 |
| 10443949 | Adamts10 | 0.763 | 9.261699 | 8.871732 |
| 10584047 | Adamts8 | 0.761 | 6.511917 | 6.118506 |
| 10554045 | Adamts17 | 0.757 | 7.834566 | 7.432868 |
| 10505717 | Adamtsl1 | 0.757 | 9.199924 | 8.79778 |
| 10500183 | Adamtsl4 | 0.752 | 9.287502 | 8.8762 |
| 10431749 | Adamts20 | 0.752 | 5.520986 | 5.109027 |
| 10531201 | Adamts3 | 0.723 | 7.820903 | 7.353565 |
| 10470349 | Adamts13 | 0.707 | 6.746606 | 6.247306 |
| 10455919 | Adamts19 | 0.7 | 6.496653 | 5.98132 |
| 10546430 | Adamts9 | 0.699 | 9.963381 | 9.447266 |
| 10591997 | Adamts8 | 0.69 | 8.166987 | 7.631823 |
| 10531166 | Adamts3 | 0.683 | 9.324503 | 8.773882 |
| 10546434 | Adamts9 | 0.68 | 9.578959 | 9.022643 |
| 10546450 | Adamts9 | 0.677 | 10.19766 | 9.634889 |
| 10546452 | Adamts9 | 0.663 | 9.863256 | 9.26956 |
| 10531191 | Adamts3 | 0.66 | 9.700072 | 9.101331 |
| 10470392 | Adamtsl2 | 0.659 | 6.895135 | 6.292624 |
| 10554074 | Adamts17 | 0.655 | 6.27718 | 5.666601 |
| 10531183 | Adamts3 | 0.653 | 8.464156 | 7.848855 |
| 10531197 | Adamts3 | 0.647 | 9.585886 | 8.957079 |
| 10531193 | Adamts3 | 0.632 | 10.41125 | 9.749179 |
| 10546454 | Adamts9 | 0.616 | 9.613199 | 8.913481 |
| 10554061 | Adamts17 | 0.615 | 8.689421 | 7.987326 |
| 10531203 | Adamts3 | 0.614 | 8.477398 | 7.77421 |
| 10531195 | Adamts3 | 0.612 | 9.371759 | 8.66361 |
| 10581961 | Adamts18 | 0.609 | 8.854218 | 8.138874 |
| 10554063 | Adamts17 | 0.606 | 7.997081 | 7.274032 |
| 10531189 | Adamts3 | 0.602 | 9.288847 | 8.556475 |
| 10505734 | Adamtsl1 | 0.596 | 9.340946 | 8.593727 |
| 10531179 | Adamts3 | 0.59 | 7.850422 | 7.089861 |
| 10554057 | Adamts17 | 0.514 | 10.05018 | 9.089145 |

Supplementary figure for Western blot

Supplementary figure 1. Western blot for figure 2C


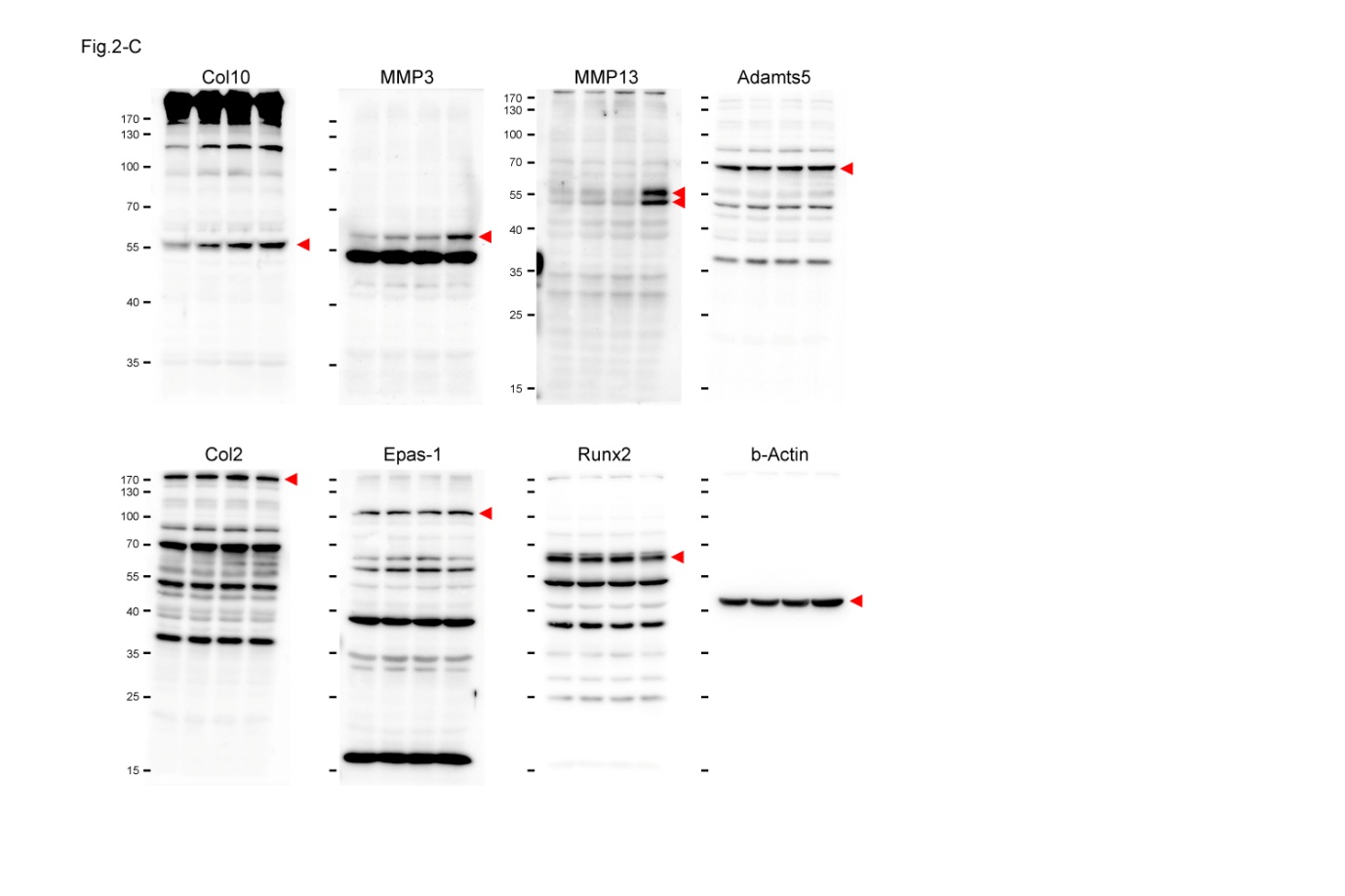


Supplementary figure 2. Western blot for figure 3A and 3B


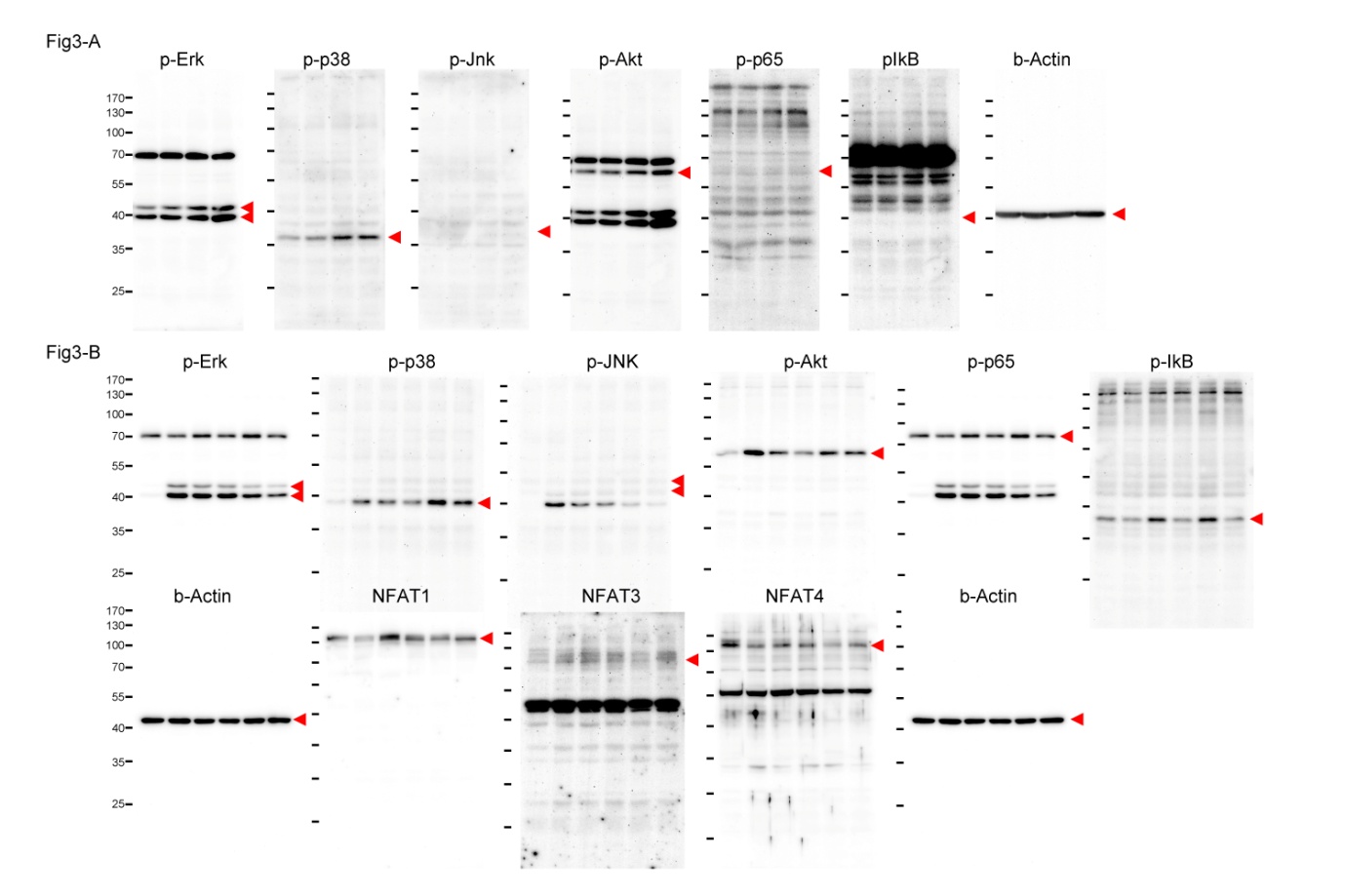


Supplementary figure 3. Western blot for figure 3C


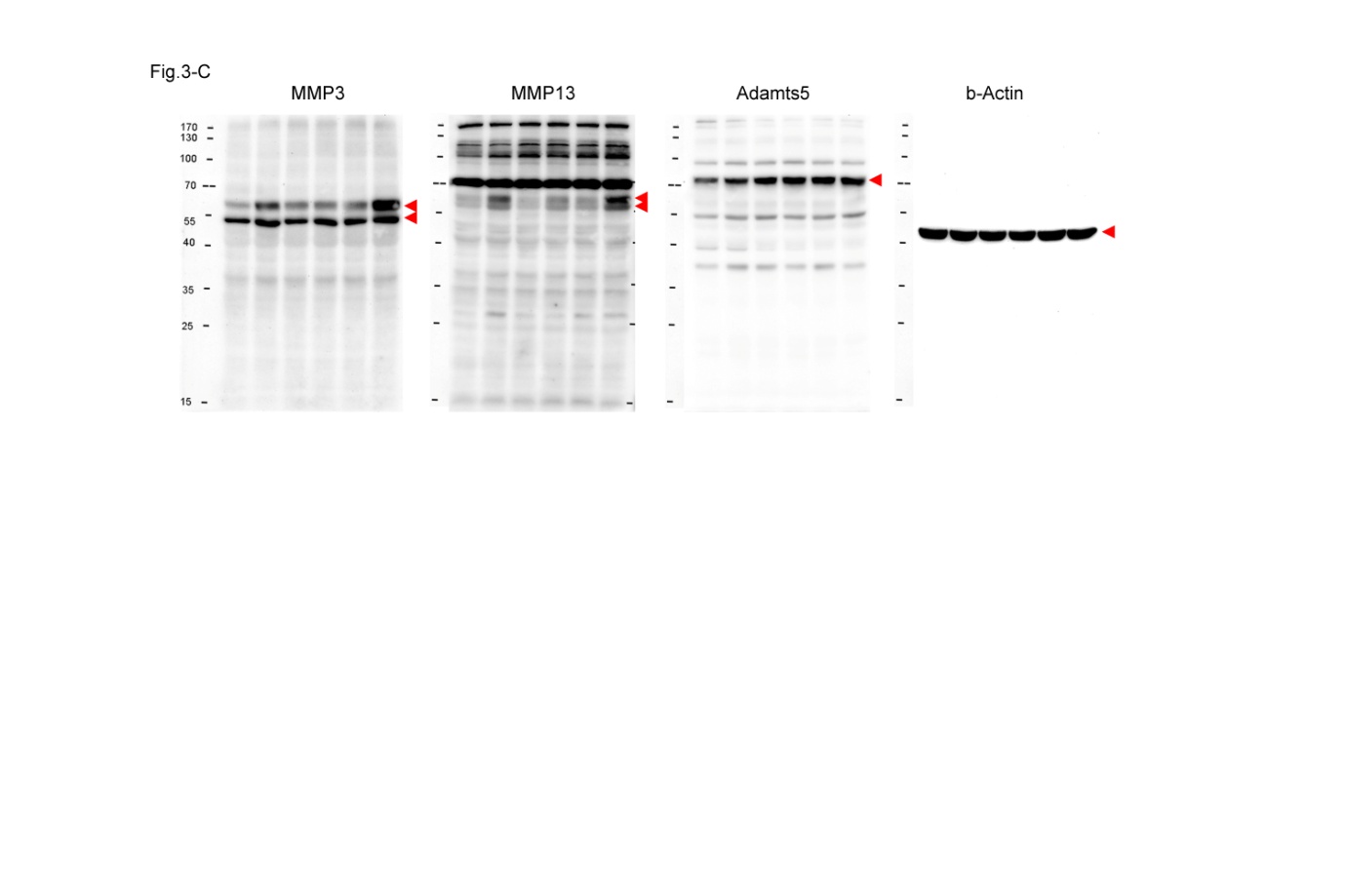


Supplementary figure 4. Western blot for figure 4A, 4C and 4E


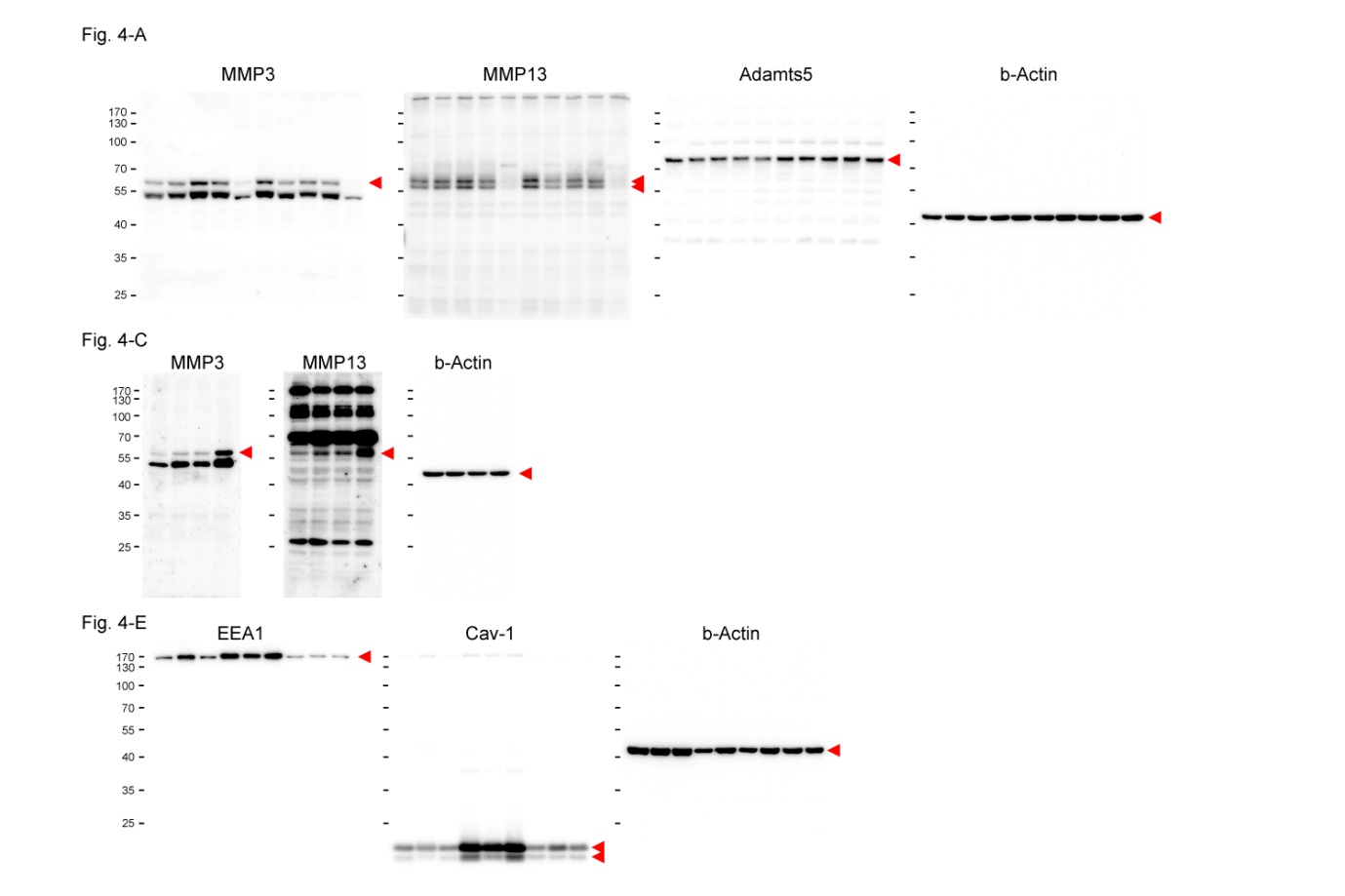


Supplementary figure 5. Western blot for figure 4F and 4G


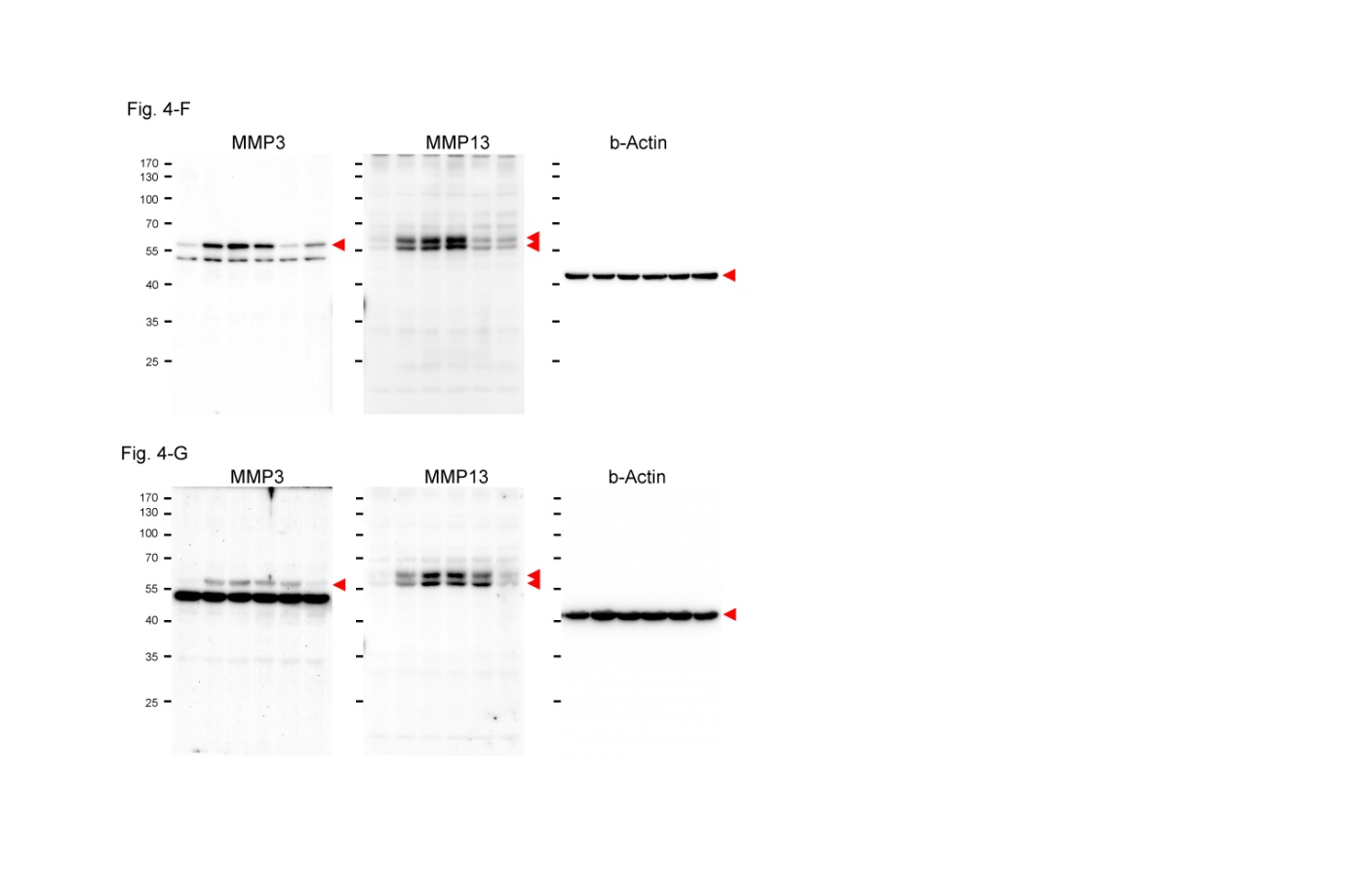


**Supplementary figure 1 for reviewer**

(A, B, C) Primary chondrocytes from E15.5 were cultured in chondrogenic media for 4 days and then treated with Ca (2.5 mM) or Pi (1.5 mM) for 1 day. Then they were harvested and subjected to real-time qPCR and Western blot analysis. (D) Representative Western blot analysis of whole cell lysates from micromass cultures treated with specific inhibitors of Erk1/2 (PD98059), p38 MAP kinase (SB203058), NF-kB (JSH-23) and calcineurin-NFAT signaling (FK506) during the co-treatment with Ca and Pi for 24 h.


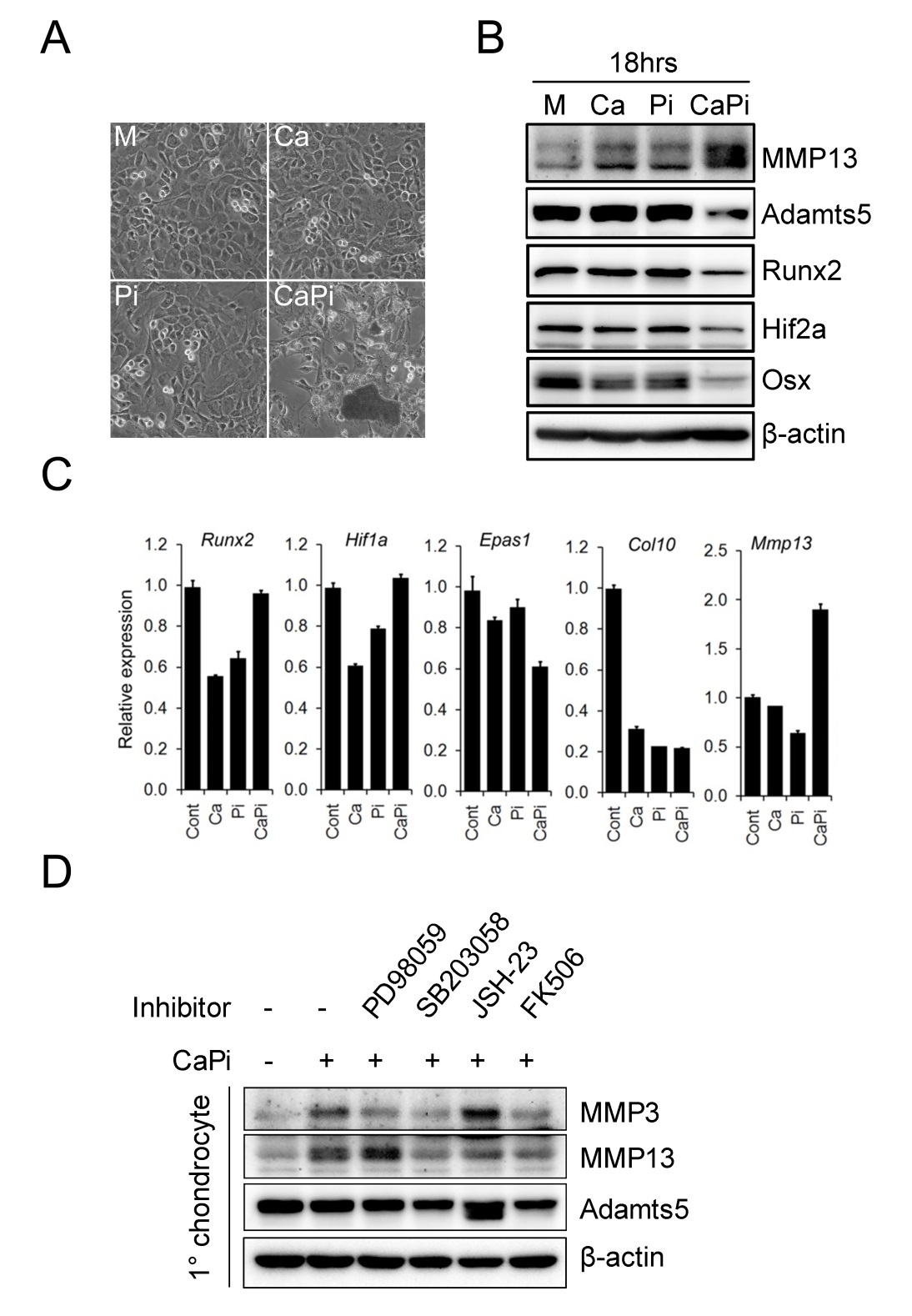

Supplement: Supplementary file 1 — Supplementary Tables and Figures [file 41598_2017_18946_MOESM1_ESM.doc]
